# Genomic profiling and drug resistance patterns of *Mycobacterium tuberculosis* in Indonesia: whole-genome sequencing of isolates from the 2017–2018 national Tuberculosis Drug Resistance Survey

**DOI:** 10.3389/fpubh.2026.1844150

**Published:** 2026-10-27

**Authors:** Yuni Rukminiati, Lamria Pangaribuan, Kindi Adam, Fauzul Muna, Dian Perwitasari, Kristina L. Tobing, Heri Wibowo, Susan Rahayu, Muhammad N. Farid, Maria Regina L. Christian, Jonathan M. M. Marbun, Andrea M. Cabibbe, Arash Ghodousi, Pratiwi P. Sudarmono, Felix Mesak, Dina Bisara Lolong

**Affiliations:** 1 National Health Biology Laboratory, Ministry of Health, Jakarta, Indonesia; 2 Doctoral Program in Biomedical Sciences, Faculty of Medicine, University of Indonesia, Jakarta, Indonesia; 3 The Health Policy Agency, Ministry of Health, Jakarta, Indonesia; 4 Directorate General of Primary and Community Health, Ministry of Health, Jakarta, Indonesia; 5 National Research and Innovation Agency, Jakarta, Indonesia; 6 Integrated Laboratory, Faculty of Medicine, University of Indonesia, Jakarta, Indonesia; 7 National Tuberculosis Expert Committee of Indonesia, Jakarta, Indonesia; 8 World Health Organization, Jakarta, Indonesia; 9 Vital Strategies, Jakarta, Indonesia; 10 IRCCS San Raffaele Scientific Institute, Milan, Italy; 11 Vita-Salute San Raffaele University, Milan, Italy

**Keywords:** tuberculosis, whole-genome sequencing, Indonesia, drug resistance, pre-XDR

## Abstract

**Objective:**

Drug-resistant tuberculosis remains a major public health challenge in Indonesia. This study aimed to characterize the genetic diversity, lineage distribution, and genotypic resistance profiles of *Mycobacterium tuberculosis* isolates across the Indonesian archipelago using whole-genome sequencing.

**Methods:**

A cross-sectional study was conducted using 1,281 analyzed MTB isolates derived from the 2017–2018 National Tuberculosis Drug Resistance Survey (DRS TB). Samples were screened via Xpert MTB/RIF and phenotypic drug susceptibility testing using the MGIT 960 system. Genomic DNA was extracted using the CTAB protocol. Whole-genome sequencing (WGS) libraries were prepared using the Nextera XT DNA Library Preparation Kit (Illumina, San Diego, CA, USA). Sequencing was performed on an Illumina MiSeq platform using the MiSeq Reagent Kit v3 (600 cycles), generating paired-end reads of 2 × 300 bp. To identify drug resistance-associated mutations and determine strain lineage/sub-lineages, raw sequencing reads were initially processed with Trimmomatic. The filtered reads were subsequently aligned to the *Mycobacterium tuberculosis* H37Rv reference genome (ATCC 27294;NC_000962.3) using the MTBseq pipeline, which the BWA-MEM algorithm (v0.7.17) for sequence mapping. Variant calling was carried out with Samtools mpileup (v1.6).

**Results:**

Four major MTB lineages were identified: Lineage 4 (43.1%) was dominant, followed by Lineage 2 (30.4%) and Lineage 1 (26.3%), while Lineage 3 was rare, with only 0.16% detected. At the sub-lineage level, L2.2.1 (18.5%), L1.2.1 (11.3%), and L4.1.2.1 (9.7%) were the most prevalent. Resistance to first-line drugs was the most frequent, primarily driven by mutations in the *katG* p.Ser315Thr (7.5%) and *rpoB* p.Ser450Leu (5%), conferring isoniazid and rifampicin resistance, respectively. Second-line resistance was highest for Fluoroquinolones (2.03%). Notably, four pre-XDR strains (0.31%) were identified and validated across Java-Bali and Sumatera, all harboring critical *gyrA* mutations and *rpoB* mutations (p.Asp435Tyr, p.Asn437Asp, p.Ser450Leu, and p.Ser450Trp).

**Conclusion:**

The study confirms a high prevalence of L2 and L4 in Indonesia. The identification of pre-XDR-TB isolates among the national survey samples highlights an urgent need for expanded molecular surveillance. While newer drugs like Bedaquiline remain effective, the presence of specific *gyrA* mutations underscores the necessity of integrating WGS into national TB control programs to proactively manage resistance and optimize treatment regimens.

## Introduction

According to the Global Tuberculosis Report 2025, 30 countries continue to bear the highest burden of TB worldwide. Indonesia is the second-highest TB burden country, after India (25%), followed by Indonesia (10%), the Philippines (6.8%), China (6.5%), Pakistan (6.3%), Nigeria (4.8%), the Democratic Republic of the Congo (3.9%), and Bangladesh (3.6%) (1). In the national context, the Indonesian National Sample Registration System (SRS) in 2001 identified TB as the third leading cause of death in the country. Furthermore, studies indicate that TB is the leading cause of mortality among infectious diseases and the second leading cause of death overall in Indonesia (2).

Drug-resistant TB (DR-TB) remains a critical public health crisis in Indonesia, with a significant portion of Rifampicin-resistant cases also being Multidrug-resistant (MDR). Based on data in 2025, there are 10,834 people suffering from drug-resistant TB and according to the WHO Global Tuberculosis Report 2025, Indonesia ranked among the five countries accounting for over half of global MDR/RR-TB incident cases (1). Moreover, between 2017 and 2022, the national tuberculosis information system (SITB) recorded 54,291 DR-TB cases out of 3,146,975 total notified TB cases, of which only 30,528 (56.2%) were enrolled on treatment underscoring persistent gaps in the DR-TB care cascade. Indonesia is formally designated by WHO as one of the 30 high MDR/RR-TB burden countries for the period 2021–2025, a list encompassing 43 countries that appear in one or both of the high TB burden and high MDR/RR-TB burden country lists. Collectively, these figures underscore the urgent need to strengthen DR-TB diagnostic capacity and surveillance for drug-resistant TB in Indonesia.

The treatment of DR-TB is significantly more time-consuming and costly, and is associated with more severe adverse effects compared to drug-susceptible tuberculosis (DS-TB). Inadequate or inappropriate treatment regimens further increases the risk of additional drug resistance and necessitate prolonged therapy. Consequently, accurate drug susceptibility testing (DST) is essential to ensure treatment efficacy and to minimize the development of further resistance.

The primary mechanism of resistance within the *Mycobacterium tuberculosis* complex (MTBC) is the acquisition of mutations in genes encoding drug targets or activating enzymes (3). In addition, drug efflux pumps represent another important mechanism contributing to increased levels of resistance (4).

Phenotypic DST often faces technical challenges such as long turnaround times and risk of contamination. Culture-based DST for *Mycobacterium tuberculosis* are laborious and technically difficult, especially for low- and middle-income countries like Indonesia, an archipelagic nation. Although pDST is the gold standard for TB diagnosis, identifying pre-XDR and XDR-TB becomes challenging on a nationwide scale. Performing pDST requires highly trained personnel, well-equipped laboratories, and appropriate biosafety infrastructure. In Indonesia, the BACTEC™ MGIT 960^®^ (Becton Dickinson, Sparks, MD, USA) is the primary platform used for culture and phenotypic drug susceptibility testing of *Mycobacterium tuberculosis*.

Molecular diagnostic techniques have gained widespread acceptance following WHO endorsement for drug-resistant tuberculosis detection. Advances in sequencing technologies, particularly Illumina-based whole-genome sequencing (WGS), enable the simultaneous identification of single nucleotide polymorphisms (SNPs) associated with resistance-conferring genes. WGS provides a high-resolution alternative by enabling comprehensive detection of chromosomal mutations and facilitating the characterization of the molecular epidemiology of TB transmission. In addition, WGS allows more accurate prediction and cross-validation of phenotypic DST results and enables detailed analysis of strain lineages and transmission dynamics. However, previous findings from Indonesia indicate variable concordance between MGIT 960^®^ and WGS, with agreement ranging from approximately 60 to 90% for first-line drugs and around 75% for second-line drugs, depending on the antibiotics tested (5).

Between 2017 and 2018, the National Institute of Health Research and Development, in collaboration with the Directorate of Disease Prevention and Control of the Ministry of Health, completed the first National Drug-Resistant Tuberculosis Survey (DRS-TB) in Indonesia. The survey revealed that the proportion of Rifampicin-resistant (RR) cases among smear-positive new cases was 2.6% (95% CI, 1.9–5.0%), while for retreatment cases, the proportion was 17.8% (95% CI, 12.5–24.7%).

Furthermore, the proportion of Multidrug-resistant TB (MDR-TB) among smear-positive new cases was found to be 1.4% (95% CI: 0.9–2.2%), and 12.4% (95% CI: 8.1–18.7%) for retreatment cases. As a follow-up to this national survey, a sub-sample of DRS-TB isolates was subjected to WGS between 2019 and 2020. The application of WGS to isolates collected through the DRS-TB survey represents a critical methodological advancement, transforming a resistance prevalence survey into a comprehensive genomic surveillance platform. This approach enables the simultaneous characterization of resistance-associated mutations across multiple drug classes, elucidates resistance mechanisms at the molecular level, and generates phylogenetic data for lineage classification and transmission analysis.

## Methods

### Study design, sample size, and WGS eligibility criteria

This study employed a cross-sectional survey design using stratified multi-stage cluster sampling based on smear microscopy–positive (acid-fast bacilli–positive) tuberculosis cases. Samples were derived from the 2017–2018 National Drug Resistance Tuberculosis Survey (DRS-TB), encompassing 40 districts/cities across 12 provinces, with 70 healthcare facilities selected to represent a nationally representative dataset of Indonesia.

Smear-positive tuberculosis cases identified from the TB06 form or laboratory logbook at healthcare facilities that met the predetermined inclusion and exclusion criteria were enrolled as study participants. Eligible participants received a thorough explanation of the study objectives and procedures, after which written informed consent was obtained. Structured interviews were subsequently conducted using an individual questionnaire. Sputum specimens were transported to reference laboratories for analysis using the Xpert^®^ MTB/RIF assay (Cepheid, Sunnyvale, CA, USA).

Prior to examination, selected sputum specimens were homogenized according to standardized laboratory protocols. The Xpert MTB/RIF assay was employed for the identification of *Mycobacterium tuberculosis* (MTB) and the detection of rifampicin resistance. The results obtained from the Xpert MTB/RIF assay were subsequently utilized as a screening tool to determine eligibility for pDST. All specimens yielding a positive MTB result on the Xpert MTB/RIF assay were subjected to culture. Phenotypic Drug Susceptibility Testing (pDST) was performed on all specimens with confirmed MTB positivity in which rifampicin resistance was detected, as well as on a randomly selected 10% of specimens that were MTB-positive however in which rifampicin resistance was not detected (rifampicin-susceptible). The anti-tuberculosis drugs included in the pDST panel comprised rifampicin, isoniazid (INH), ethambutol, streptomycin, amikacin, kanamycin, and ofloxacin. Culture and pDST were performed using the BD BACTEC™ MGIT 960^®^ system (Becton, Dickinson and Company, Franklin Lakes, NJ, USA). Following culture, all isolates were stored at −80 °C until further analysis.

Specimens selected for whole-genome sequencing (WGS) comprised all specimens with Xpert MTB/RIF rifampicin-resistant results (*n* = 161); one of these was subsequently excluded because of culture failure, leaving 160 specimens available for WGS. In addition, the following rifampicin-sensitive (RS-TB) specimens were selected: all retreatment cases that had undergone phenotypic drug susceptibility testing (pDST) (*n* = 43); all retreatment cases that had not undergone pDST (*n* = 415), two of which were subsequently excluded because of culture failure, leaving 413 specimens; all new cases that had undergone pDST (*n* = 241); and 35% of new cases that had not undergone pDST (*n* = 741). Altogether, this yielded 1,598 eligible specimens for WGS. Of these, 63 were excluded because of culture failure, so DNA extraction was attempted on 1,535 specimens. DNA extraction and library preparation were successful for 1,429 specimens; the remaining 106 were excluded because of insufficient DNA quantity (<1.7). Following sequencing, 148 specimens did not meet the pre defined WGS quality-control criteria (depth < 30× and coverage < 90%) and were excluded. Ultimately, 1,281 specimens (80.2%) were successfully processed and yielded analyzable WGS data (Figure 1).

**Figure 1:**
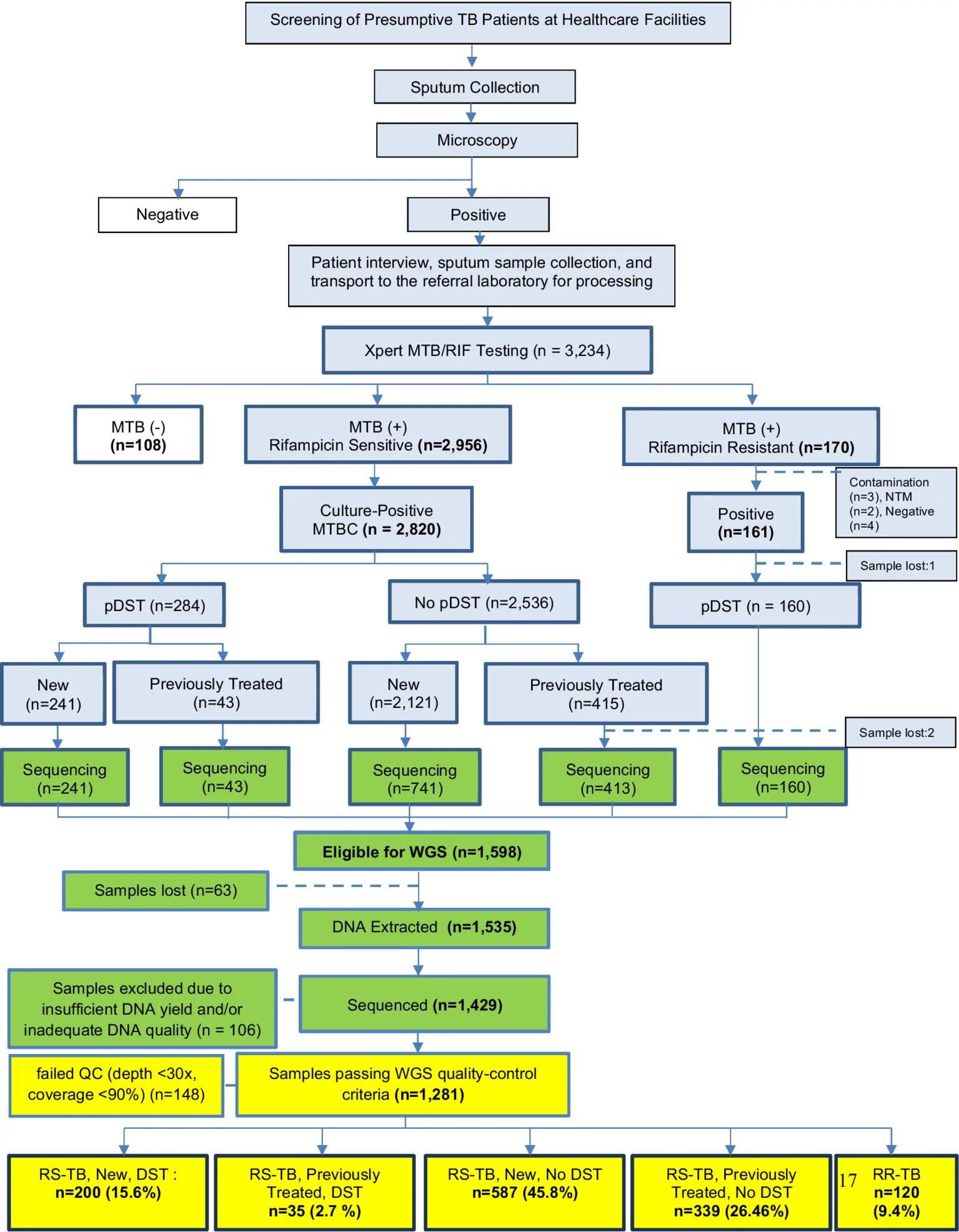
Diagram of Whole-Genome Sequensing selection results. MTB, *Mycobacterium tuberculosis*; DST, drug susceptibility testing; NTM, Nontuberculous Mycobacteria; RS-TB, rifampicin-sensitive tuberculosis; RR-TB, rifampicin-resistant tuberculosis.

### Reculture and isolate preparation

Reculture of stored isolates was performed at three designated pDST reference laboratories across Indonesia. Following successful culture growth, isolates were heat-inactivated at 95 °C for 30 min in a heat block to ensure biosafety prior to transport. Inactivated isolates were subsequently shipped to two sequencing facilities: the National Laboratory of Prof. Sri Oemijati, Ministry of Health of the Republic of Indonesia, and the Integrated Laboratory of the Faculty of Medicine, Universitas Indonesia (FKUI), where whole-genome sequencing was carried out.

### Genomic DNA extraction, library preparation and whole-genome sequencing

Total DNA extraction, isolation, and purification of MTBC bacteria were accomplished using the N-cetyl-N, N, N-trimethyl ammonium bromide (CTAB). Afterward, Qubit Fluorometer 4.0 and Nanodrop (Thermo Scientific, Waltham, MA, USA) were used to measure the quantity and quality of the isolated DNA, respectively. For preparing the next whole-genome sequence, the limit of DNA quantity and purity is set to ≥0.2 ng/μL and 1.7 ≥ A_260_/A_280_ ≤ 2.0, respectively.

Whole-genome sequencing libraries were prepared using the Nextera XT DNA Library Preparation Kit (Illumina, San Diego, CA, USA). Sequencing was performed on an Illumina MiSeq platform using the MiSeq Reagent Kit v3 (600-cycle), generating paired-end reads of 2 × 300 bp, according to the manufacturer’s instructions.

Library preparation on the MiSeq platform involved the following steps: (1) genomic DNA tagmentation, (2) library amplification, (3) library clean-up, (4) library pooling, and (5) loading onto the reagent cartridge. Prior to loading, libraries were normalized and quantified using the KAPA Library Quantification Kit for Illumina platforms (Roche, Basel, Switzerland), following the manufacturer’s recommendations.

### Bioinformatics analysis

To identify drug resistance–associated mutations and determine strain lineage/sublineages, raw sequencing reads were initially processed with Trimmomatic (6) to remove adapter sequences, and reads shorter than 20 bp were discarded. The filtered reads were subsequently aligned to the *Mycobacterium tuberculosis* H37Rv reference genome (ATCC 27294; NC_000962.3) using the MTBseq pipeline (7), which applies the BWA-MEM algorithm (v0.7.17) (8) for sequence mapping. Duplicate reads were marked with Picard (v2.23.4–0), and local realignment around insertion–deletion (InDel) regions was performed using the Genome Analysis Toolkit (GATK v3.8). Variant calling was carried out with Samtools mpileup (v1.6) (9) using a minimum mapping quality of 20, a minimum base quality of 20, a minimum read depth of 8×, and a maximum strand bias of 75%. To enable the detection of potential resistant subpopulations, MTBseq parameters were further optimized for low-frequency variant identification (7), allowing the detection of mutations supported by at least two sequencing reads and present at a minimum allele frequency of 5%.

### Statistical analysis

Analysis was performed using Stata version 15.0 (StataCorp, College Station, Texas, USA) software. The proportion of resistance to each antibiotic was estimated separately for new and previously treated tuberculosis cases. Given the complexity of the national survey sampling design which employed stratified cluster sampling with probability-proportional-to-size selection, all analyses incorporated survey probability weights to ensure nationally representative estimates and to correct for disproportionate sampling across strata.

Point estimates of resistance proportions (%) are presented alongside their standard errors (SE), which were derived using Taylor series linearization to appropriately account for design effects. This approach yields more accurate variance estimates than those produced under simple random sampling assumptions, particularly in settings with hierarchical or clustered data structures. Ninety-five percent confidence intervals (95% CI) were constructed using the logit transformation method.

### Data availability

WGS data for four pre-XDR *M. tuberculosis* strains and two Lineage 3 strains are available in the NCBI Sequence Read Archive (SRA) under BioProject accession number PRJNA950554.

**Table:** 

Accession Number	Title	BioSample	Library ID
SRR29659248	WGS of *Mycobacterium tuberculosis*: culture	SAMN42182043	R3619
SRR29659249	WGS of *Mycobacterium tuberculosis*: culture	SAMN42182042	R3618
SRR29659247	WGS of *Mycobacterium tuberculosis*: culture	SAMN42182044	R737
SRR29659250	WGS of *Mycobacterium tuberculosis*: culture	SAMN42182041	R475
SRR24682255	WGS of *Mycobacterium tuberculosis*	SAMN35301034	S6970
SRR24682254	WGS of *Mycobacterium tuberculosis*	SAMN35082234	S19106

## Results

### Characteristics of the study population

Table 1 presents the socioeconomic and demographic characteristics of the study sample, stratified by case classification into new cases and retreatment (recurrent) cases. The majority of participants in both groups fell within the productive age range. When stratified by age group, the proportion of new cases was notably higher among those aged under 15 years and those in the 15–24 year cohort; conversely, retreatment cases predominated across all remaining age groups.

**Table 1:** Demographic, socioeconomic, and clinical characteristics of the study population.

Characteristic	New cases (*n* = 836)	Retreated (*n* = 445)	Total (*n* = 1,281)
	*n* (%)	*n* (%)	*n* (%)
Age group (years)			
<15	2 (0.24)	0 (0.00)	2 (0.16)
15–24	155 (18.54)	54 (12.13)	209 (16.32)
25–34	160 (19.14)	89 (20.00)	249 (19.44)
35–44	156 (18.66)	97 (21.80)	253 (19.75)
45–54	175 (20.93)	101 (22.70)	276 (21.55)
55–64	128 (15.31)	70 (15.73)	198 (15.46)
65+	60 (7.18)	34 (7.64)	94 (7.34)
Gender			
Male	524 (62.68)	298 (66.97)	822 (64.17)
Female	312 (37.32)	147 (33.03)	459 (35.83)
Education			
Never schooled/Did not finish Elementary	105 (12.56)	51 (11.46)	156 (12.18)
Finished Elementary School	225 (26.91)	125 (28.09)	350 (27.32)
Finished Junior High School	185 (22.13)	114 (25.62)	299 (23.34)
Finished Senior High School/University	321 (38.40)	155 (34.83)	476 (37.16)
Employment status			
Employed	445 (53.23)	246 (55.28)	691 (53.94)
Unemployed	391 (46.77)	199 (44.72)	590 (46.06)
If employed, primary occupation			
Government/Private Employee	98 (22.02)	41 (16.67)	139 (20.12)
Entrepreneur/Self-employed	122 (27.42)	81 (32.93)	203 (29.38)
Fisherman/Farmer/Laborer	207 (46.52)	106 (43.09)	313 (45.30)
Others	18 (4.04)	18 (7.32)	36 (5.21)
Region			
Sumatra	295 (35.29)	145 (32.58)	440 (34.35)
Java-Bali	347 (41.51)	193 (43.37)	540 (42.15)
Eastern Indonesia (other)	194 (23.21)	107 (24.04)	301 (23.50)

With respect to sex distribution, male participants constituted approximately twice the number of female participants. Within the male subgroup, the proportion of retreatment cases was higher, whereas new cases were more frequently observed among female participants.

Regarding educational attainment, the largest proportion of the study sample had completed Senior High School or attained a University-level education. Comparative analysis demonstrated that retreatment cases were more prevalent among individuals with Elementary or Junior High School education, while new cases were more commonly observed among those with higher educational attainment.

Analysis of employment status revealed that the majority of participants were currently employed. When stratified by clinical classification, the proportion of retreatment cases was higher among employed individuals, whereas new cases were more frequent among those who were unemployed.

Among the primary occupations reported by employed participants, the fisherman/farmer/laborer category represented the largest occupational group. Retreatment cases were proportionately higher among entrepreneurs and self-employed individuals, as well as those classified under other occupational categories, whereas new cases were more prevalent among office employees and fishermen/farmers/laborers.

Geographically, the largest proportion of the study sample was drawn from the Java-Bali region relative to the other two regions. Notably, retreatment cases showed a higher concentration in Java-Bali, while new cases were more frequently observed in Sumatra and the Eastern Indonesia regions.

The results of Xpert MTB/RIF show in Figure 2, the map illustrates the distribution of tuberculosis samples DRS, categorized into two groups: rifampicin-sensitive TB (RS-TB, shown in green) and rifampicin-resistant TB (RR-TB, shown in red). The largest number of samples was collected from DKI Jakarta (155 RS-TB, 17 RR-TB) and Jawa Tengah (138 RS-TB, 9 RR-TB), reflecting their status as major population centers with high TB burdens. Sumatera Utara also contributed a substantial number of samples (141 RS-TB, 21 RR-TB), recording the highest absolute count of RR-TB cases among all provinces included.

**Figure 2:**
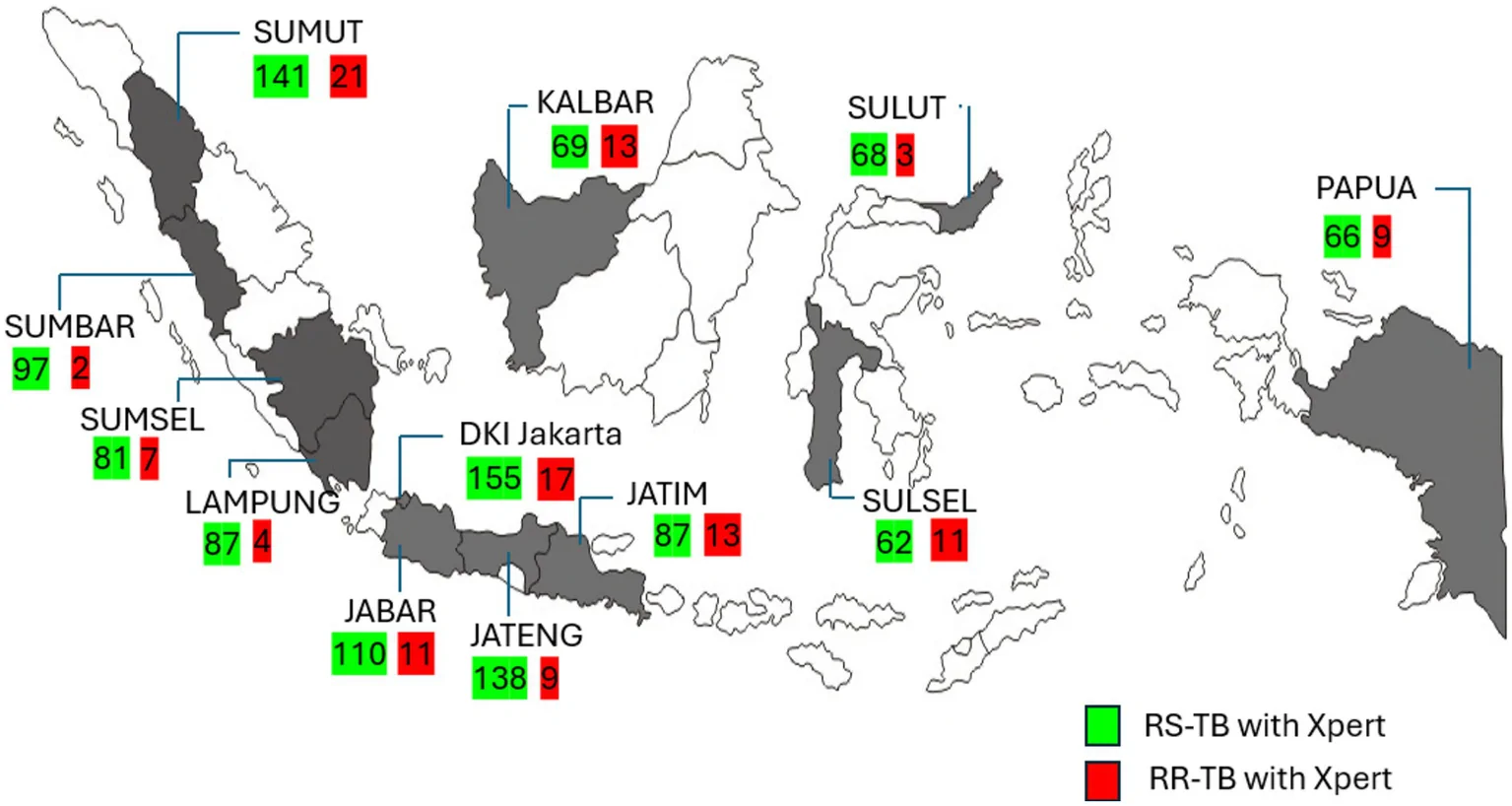
Geographic distribution of rifampicin-sensitive tuberculosis (RS-TB) and rifampicin resistant tuberculosis (RR-TB) samples detected by Xpert MTB/RIF.

### Genetic diversity and distribution of MTB lineages in Indonesia

Table 2 presents the distribution of *Mycobacterium tuberculosis* genetic lineages identified in Indonesia, classified into four major lineages: Lineage 1, lineage 2, lineage 3, and lineage 4.

**Table 2:** Identification of MTB lineage results from WGS examination according to Coll et al. (3).

Lineage	Sub-lineage	Total
1 (*n* = 337)_	1	6
	1.1.1	92
	1.1.2	4
	1.1.3	33
	1.2.1	145
	1.2.2	57
2 (*n* = 396)	2	-
	2.1	45
	2.2	2
	2.2.1	237
	2.2.1.1	59
	2.2.1.2	14
	2.2.2	33
3 (*n* = 2)	3	2
4 (*n* = 546)	4	4
	4.1.1	2
	4.1.1.1	1
	4.1.1.3	4
	4.1.2	13
	4.1.2.1	124
	4.2.2	5
	4.3	4
	4.3.1	102
	4.3.2	4
	4.3.3	45
	4.3.4.1	1
	4.3.4.2	4
	4.4.1	5
	4.4.1.1	44
	4.4.1.2	34
	4.4.2	22
	4.5	40
	4.7	16
	4.8	66
	4.9	12

Lineage 4 was identified as the dominant lineage among the examined specimens (546 (43%)), followed by Lineage 2 (396 (30.91%)).

Lineages 1 and 3 were found to exhibit a geographically restricted distribution, localized predominantly to East Africa, South Asia, and Southeast Asia; notably, Lineage 3 was detected at very low frequencies within the study population and the results of this study are the first report of TB sequencing results for the presence of lineage 3 strains in Indonesia.

The most prevalent sub-lineage identified was the sub-lineage 2.2.1 and its variants, comprising a total of 18.50% isolates. The second most frequently identified group was sub-lineage 1.2.1, with 11.31% isolates. The third most common sub-lineage was the sub-lineage 4.1.2.1, accounting for 9.6% isolates.

### Genotypic resistance profiles of first- and second-line anti-tuberculosis drugs

Table 3 shows the whole-genome sequencing (WGS) profile for each drug resistance-associated gene detectable by the Mtbseq pipeline. For each antibiotic and its associated gene(s), raw (unweighted) values are reported prior to any statistical weighting or adjustments.

**Table 3:** Antibiotic genetic resistance profile.

Antibiotic	Gene	Sensitive	Resistant	Observed
		*n* (%)	*n* (%)	*n* (%)
Rifampicin, Rifabutin	*rpoB*	505 (39.42)	136 (10.62)	640 (49.96)
	*rpoC*			
Compensates for KatG deficit	*ahpC*	1,106 (86.34)	0 (0)	175 (13.66)
Isoniazid	*furA*	1,281 (100)	0 (0)	0 (0)
	*katG*	652 (50.90)	101 (7.88)	528 (41.22)
	*fabG1*	909 (70.96)	0 (0)	372 (29.04)
	*inhA*	908 (70.88)	5 (0.39)	368 (28.73)
Ethambutol	*embA*	567 (44.26)	3 (0.23)	711 (55.50)
	*embB*	573 (44.73)	59 (4.61)	649 (50.66)
Pyrazinamide	*pncA*	1,084 (84.62)	35 (2.73)	162 (12.65)
	*rpsA*	845 (65.96)	2 (0.16)	434 (33.88)
Streptomycin	*Gid*	1,016 (79.31)	5 (0.39)	260 (20.30)
	*rpsL*	1,123 (87.67)	31 (2.42*)	127 (9.91)
	*Rrs*	412 (32.16)	20 (1.56)	849 (66.28)
Kanamycin	*rrs*	412 (32.16)	20 (1.56)	849 (66.28)
Amikacin	*rrs*	412 (32.16)	20 (1.56)	849 (66.28)
Ofloxacin	*gyrA*	1,258 (98.20)	23 (1.80)	0 (0)
	*gyrB*	856 (66.82)	4 (0.31)	421 (32.86)
Moxifloxacin	*gyrA*	1,258 (98.20)	23 (1.80)	0 (0)
	*gyrB*	856 (66.82)	4 (0.31)	421 (32.86)
Levofloxacin	*gyrA*	1,258 (98.20)	23 (1.80)	0 (0)
	*gyrB*	856 (66.82)	4 (0.31)	421 (32.86)
Bedaquiline	*atpE, pepQ, Rv0678, Rv1979c*	1,281 (100)	0 (0)	0 (0)
Delamanid	*ddn, fbiA, fbiB, fbiC, fgd1*	1,281 (100)	0 (0)	0 (0)
Linezolid	*rplC*	1,085 (84.70)	0 (0)	196 (15.30)
	*rrl*	193 (15.07)	1 (0.08)	1,087 (84.86)
Capreomycin	*tlyA*	1,281 (100)	0 (0)	0 (0)
	*rrl*	193 (15.07)	1 (0.08)	1,087 (84.86)
	*rrs*	412 (32.16)	20 (1.56)	849 (66.28)
Clofazimine	*pepQ, Rv0678, Rv1979c*	1,281 (100)	0 (0)	0 (0)
Ethionamide	*fabG1*	909 (70.96)	0 (0)	372 (29.04)
	*inhA*	908 (70.88)	5 (0.39)	368 (28.73)
	*ethA*	908 (70.88)	1 (0.08)	372 (29.04)
Prothionamide	*fabG1*	909 (70.96)	0 (0)	372 (29.04)
	*inhA*	908 (70.88)	5 (0.39)	368 (28.73)
	*ethA*	908 (70.88)	1 (0.08)	372 (29.04)
Clarithromycin	*rrl*	193 (15.07)	1 (0.08)	1,087 (84.86)
Gatifloxacin	*gyrA*	1,258 (98.20)	23 (1.80)	0 (0)
p-aminosalicylic acid	*thyA*	1,281 (100)	0 (0)	0 (0)
Rifabutin	*rpoB*	505 (39.42)	136 (10.62)	640 (49.96)
Thioacetazone	*ethA*	908 (70.88)	1 (0.08)	372 (29.04)

A total of 106 specimens exhibited resistance to isoniazid, with mutations in the *katG* gene (101 specimens) representing the most predominant molecular mechanism underlying this resistance followed by *inhA* mutations, which were identified in 5 specimens (Table 3). The most common mutation was *katG* p. Ser315Thr. Rifampicin resistance was predominantly mediated by mutations within the *rpoB* gene (codons 426–452), which encodes the beta-subunit of RNA polymerase. The most clinically significant substitution was *rpoB* p. Ser450Leu. Importantly, two compensatory mutations were detected in *rpoC* (p. Ile885Val and p. Leu527Val).

Resistance to second-line anti-tuberculosis agents was most frequently observed for ethionamide and prothionamide, each accounting for 3.9% of isolates. This was followed by the fluoroquinolone class, in which 26 specimens demonstrated resistance, predominantly attributable to mutations in the *gyrA* gene (n = 23; 1.8%), the most substitution was *gyrA* p. Asp94Ala. Resistance to aminoglycosides, specifically amikacin and kanamycin, was detected in 20 specimens (1.56%), mostly mutation in rrs r.1401a > g. Notably, no resistance was identified for bedaquiline, delamanid, or para-aminosalicylic acid (PAS) among the tested isolates.

### Resistance proportions for first- and second-line anti-tuberculosis drugs

Among first-line agents, isoniazid (INH) exhibited the highest resistance proportion in this study, recorded at 9.51% (95% CI: 7.79–11.57) among new cases and 17.84% (95% CI: 14.38–21.92) among retreated cases. Rifampicin (RIF) resistance was similarly elevated at 7.90% (95% CI: 6.13–10.13) among new cases and 16.70% (95% CI: 13.18–20.94) among retreated cases (Table 4).

**Table 4:** Proportion of drug resistance based on treatment history.

Antibiotic	Case type	Proportion (%)	Standard error	95% CI
Rifampicin	New cases	7.9	0.88	6.13–10.13
	Retreated cases	16.7	1.71	13.18–20.94
Isoniazid	New cases	9.51	0.83	7.79–11.57
	Retreated cases	17.84	1.66	14.38–21.92
Pyrazinamide	New cases	2.53	0.58	1.50–4.23
	Retreated cases	3.2	0.82	1.79–5.66
Ethambutol	New cases	5.06	0.89	3.38–7.50
	Retreated cases	5.32	1.2	3.16–8.8
Streptomycin	New cases	3.37	0.52	2.37–4.75
	Retreated cases	5.48	0.87	3.82–7.81
Kanamycin	New cases	0.86	0.3	0.38–1.90
	Retreated cases	1.11	0.49	0.40–3.01
Amikacin	New cases	0.86	0.3	0.38–1.90
	Retreated cases	1.11	0.49	0.40–3.01
Ofloxacin	New cases	1.67	0.28	1.14–2.44
	Retreated cases	2.79	0.76	1.49–5.14
Thioacetazone	New cases	0.12	0.08	0.03–0.51
	Retreated cases	0	0	0
Bedaquiline	New cases	0	0	0
	Retreated cases	0	0	0
Ethionamide	New cases	0.74	0.27	0.32–1.70
	Retreated cases	0	0	0
Moxifloxacin	New cases	1.78	0.31	1.19–2.64
	Retreated cases	2.76	0.75	1.49–5.07
Gatifloxacin	New cases	1.78	0.31	1.19–2.64
	Retreated cases	2.76	0.75	1.49–5.07
Levofloxacin	New cases	1.78	0.31	1.19–2.64
	Retreated cases	2.76	0.75	1.49–5.07
Prothionamide	New cases	3.26	0.56	2.20–4.81
	Retreated cases	4.86	0.74	3.43–6.85
Linezolid	New cases	0.17	0.17	0.02–1.57
	Retreated cases	0	0	0
Clarithromycin	New cases	0.17	0.17	0.02–1.57
	Retreated cases	0	0	0
Para-aminosalicylic acid	New cases	0	0	0
	Retreated cases	0	0	0
Capreomycin	New cases	1.66	0.54	0.79–3.46
	Retreated cases	1.99	0.48	1.15–3.43

Resistance to ethambutol (EMB) was observed at 5.06% (95% CI: 3.38–7.50) in new cases and 5.32% (95% CI: 3.16–8.80) in retreated cases, while streptomycin- an injectable aminoglycoside historically used as part of retreatment regimens exhibited resistance proportions of 3.37 and 5.48% in new and retreated cases, respectively. Pyrazinamide (PZA) resistance, though comparatively lower at 2.53% in new cases and 3.20% in retreated cases.

Among second-line agents, fluoroquinolones including ofloxacin, moxifloxacin, gatifloxacin, and levofloxacin, all demonstrated identical resistance proportions of 1.67–1.78% in new cases and 2.76–2.79% in retreated cases.

### Pre-XDR-TB patients

In the present study, four pre-extensively drug-resistant tuberculosis (pre-XDR-TB) strains designated Mtb_R475, Mtb_R737, Mtb_R3618, and Mtb_R3619 were identified among 1,281 isolates retrieved from the DRS-TB repository, which were collected across the Indonesian archipelago. According to WHO, pre-XDR-TB is TB caused by *Mycobacterium tuberculosis (M. tuberculosis)* strains that fulfil the definition of multidrug-resistant and rifampicin-resistant TB (MDR/RR-TB) and which are also resistant to any fluoroquinolone. Collectively, these four pre-XDR-TB strains accounted for 1.1% of the total study sample (Table 5). Confirmatory validation of the four pre-XDR-TB strains was performed using the BACTEC™ MGIT 960^®^ system and Illumina-based whole-genome sequencing (WGS).

**Table 5:** Characteristics of Pre XDR TB samples.

Attributes	Mtb_R737	Mtb_R475	Mtb_R3618	Mtb_R3619
SRA	SRR29659247	SRR29659250	SRR29659249	SRR29659248
Case status	Retreated	New	Retreated	New
Gender	Female	Male	Male	Female
Age group	35–44	25–34	35–44	15–24
Smoking status	Never smoked	Never smoked	Stopped smoking	Never smoked
Work status	Unemployed	Self-employed	Unemployed	Unemployed
Region	Java-Bali	Java-Bali	Sumatera	Sumatera
Lineage	2	4	4	4
Sub-lineage	2.2.1	4.5	4.5	4.5
Resistance mutation				
Rifampicin	*rpoB* p. Ser450Trp	*rpoB* p. Asp435Tyr	*rpoB* p. Asp435Tyr	*rpoB* p. Asp435Tyr
		*rpoB* p. Ser450Leu	*rpoB* p. Asn437Asp	*rpoB* p. Asn437Asp
			*rpoB* p. Ser450Leu	*rpoB* p. Ser450Leu
Pyrazinamid	*pncA* p. Asp12Ala			
Isoniazid	*ahpC* c.-74G > A	*katG* p. Ser315Thr	*katG* p. Ser315Thr	*katG* p. Ser315Thr
Ethambutol	*embB* p. Gly406Cys			
Fluoroquinolones	*gyrA* p. Asp94Gly	*gyrA* p. Asp94Ala	*gyrA* p. Asp94Ala	*gyrA* p. Asp94Ala

## Discussion

The distribution of *Mycobacterium tuberculosis* lineages across the Indonesian archipelago reveals a marked geographic heterogeneity, reflecting the complex interplay of historical migration, trade routes, and population dynamics. As illustrated by the pie charts overlaid on three distinct regions Sumatra, Java, and the eastern Indonesia the predominant lineages differ substantially across these areas, suggesting that multiple independent transmission networks may coexist within a single national setting (Figure 3).

**Figure 3:**
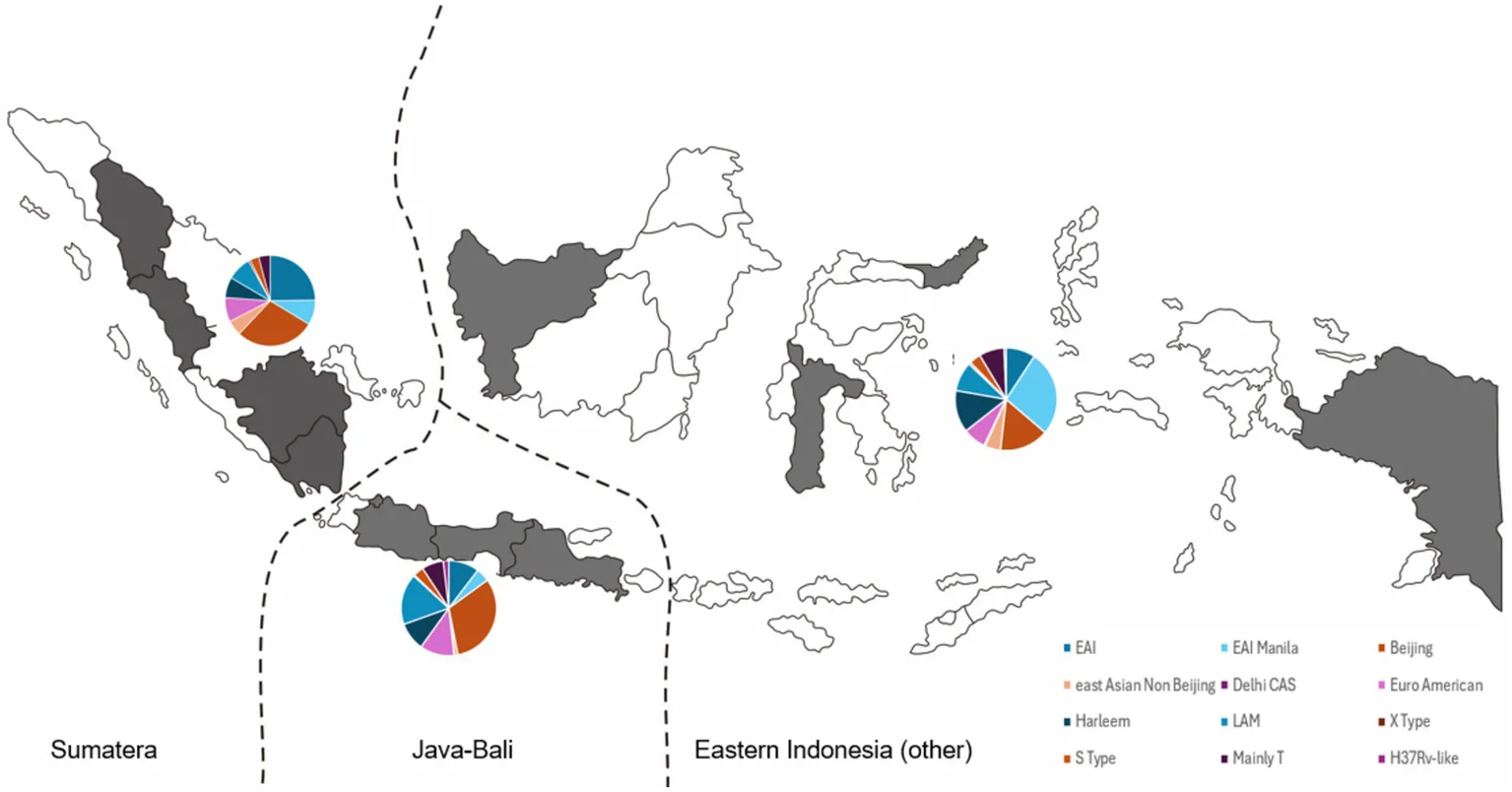
Whole-genome sequencing based phylogeographic distribution of 1,281 *Mycobacterium tuberculosis* (MTB) strains by region in Indonesia, TB lineage distribution.

Of the seven globally recognized lineages, four were identified in this research. Lineage 2 (L2) and Lineage 4 (L4) are the dominant lineages found worldwide, including in Indonesia (10, 11). Overall, L4 was the most prevalent, consistent with its global dominance. This prevalence is likely attributed to superior virulence and host adaptation factors in L4, followed by L2, enabling more rapid transmission (12). Indirectly, this suggests that L4 and L2 possess higher mutation rates, ultimately resulting in better adaptation to antibiotic pressure. Consequently, drug-resistance mutations are more frequently observed within the MTB L4 and L2 lineages (13, 14).

In the Sumatra region, the East African-Indian (EAI) lineage constitutes the dominant strain type, consistent with the well-established predominance of EAI across Southeast Asian countries (11, 15). This pattern is likely attributable to centuries of historical trade and population movement across the Bay of Bengal and the Strait of Malacca, which facilitated the spread of EAI strains throughout maritime Southeast Asia. The EAI lineage is phylogeographically associated with the eastern coast of Africa, South India, and Southeast Asia, reflecting a North–South divide along the tropic of cancer that has shaped the distribution of this lineage across the Eastern hemisphere (15). The relatively limited diversity observed in Sumatra may also reflect more geographically contained local transmission networks compared to more densely populated regions of Indonesia.

In contrast, Java-Bali the most densely populated region in Indonesia displays a considerably more heterogeneous lineage composition, with multiple strains including EAI, Beijing, Euro American (Haarlem, LAM, X-type, T-type), and EAI Manila co-circulating simultaneously. This high degree of lineage diversity is consistent with Java’s role as the nation’s demographic and economic center, where intensive internal migration, high population density, and frequent international connectivity converge to facilitate the introduction and sustained transmission of diverse *M. tuberculosis* strains (16, 17). A previous study by Parwati et al. (17) demonstrated that the Beijing genotype family accounted for 33.0% of isolates from West Java, a significantly higher proportion compared to eastern Indonesia region underscoring the elevated diversity and distinct lineage dynamics of Java relative to other regions. The presence of the Beijing lineage in Java-Bali warrants particular attention given its well-documented association with drug resistance and increased transmissibility reported across multiple epidemiological studies (18, 19).

Notably, the eastern Indonesia region demonstrates a distinct lineage profile, with a higher relative proportion of Euro American strains compared to the Sumatera regions (western). Molecular and genomic evidence indicates that Lineage 4 (L4) originated in Europe and was subsequently disseminated globally through trade routes and colonial migration (20, 21). The eastward shift in lineage distribution observed in this study may therefore reflect historical influences stemming from Dutch colonial-era trade networks, as well as the geographic positioning of these region along historical spice trade routes that connected Southeast Asia with South Asia, the Middle East, and Europe. The heterogeneous strain composition recorded in Makassar, South Sulawesi, situated along the transitional zone between western and eastern Indonesia which included EAI (30.2%), T-type (17.9%), Haarlem (12.3%), and Beijing (9.5%) lineages, further supports the existence of diverse transmission pathways across the eastern archipelago (22).

Throughout this study, unrelated Indonesian L3 strain are presented here for the first time, identified through whole-genome sequencing among 1,281 isolates from two geographically distant islands, from Central Java and South Sulawesi separated by approximately 1,400 km. Although both Indonesian L3 isolates remain susceptible to all first- and second-line anti-tuberculosis drugs, the discovery of L3 in Central Java and South Sulawesi adds a new dimension to the distribution map of Indonesian lineages that in addition to L1, L2, and L4, L3 now also needs to be considered as a lineage that is currently entering Indonesia, possibly through international travel transmission (23).

The emergence of Antituberculosis drugs resistance is a significant barrier to treatment efficacy. Resistance can occur naturally through spontaneous chromosomal mutations (24). However, inadequate treatment further drives this phenomenon. Generally, MTB resistance is caused by chromosomal mutations via mechanisms such as drug target evasion, target over-expression, interference with drug activation, and the activation of efflux pump systems.

Resistance pathways are categorized into primary resistance (exposure to an already resistant strain) and secondary (acquired) resistance (resulting from improper treatment or poor-quality drugs). Prevention relies on infection control, environmental intervention, host immune system enhancement, treatment of latent TB, accurate diagnosis, and robust patient support during therapy.

Among first-line agents, INH resistance exceeding RIF resistance in both new and retreated cases mirrors the global trend, in which isoniazid mono-resistance is generally more prevalent than rifampicin resistance and is frequently under-detected because rapid molecular diagnostics such as Xpert MTB/RIF primarily target rifampicin resistance, leaving INH resistance dependent on phenotypic or line-probe assays that are less consistently available (25). The markedly higher resistance among retreated cases compared with new cases replicates the well-established global finding that a history of prior TB treatment is the strongest risk factor for drug resistance, reflecting selective pressure from incomplete adherence, inadequate regimens, or interrupted therapy (1).

In this study, mutations were detected in *rpoC*. While mutations in the *rpoB* gene, encoding the β-subunit of bacterial RNA polymerase, are the primary determinants of rifampicin resistance, they frequently impose substantial fitness costs that compromise bacterial transmissibility and persistence. Secondary compensatory mutations in the *rpoC* gene, encoding the β’-subunit, represent well-established molecular signatures of clonal adaptation that arise in response to these fitness deficits. Mutations such as p. Ile885Val and p. Leu527Val, located within the interaction interface between rpoB and rpoC subunits, functionally restore RNA polymerase activity and bacterial fitness by restabilizing the transcriptional machinery and normalizing transcription kinetics (26).

The high proportions of isoniazid and rifampicin resistance observed in both case types are of particular clinical concern, as concurrent resistance to both agents the definition of multidrug-resistant tuberculosis (MDR-TB) renders first-line short-course chemotherapy ineffective and necessitates the use of longer, more toxic, and more costly treatment regimens. Indonesia is among the countries with the highest estimated MDR-TB burden globally, and the resistance proportions identified here are consistent with this epidemiological profile. Pyrazinamide (PZA) resistance in this study is noteworthy given that pyrazinamide plays a critical role in sterilizing activity during the first 2 months of standard treatment and is a key component of many shorter MDR-TB regimens. The relatively narrow difference in ethambutol and pyrazinamide resistance between new and retreated cases may suggest that resistance to these agents arises independently of prior treatment history, potentially reflecting primary transmission of resistant strains within the community.

Currently, phenotypic DST is limited to antituberculosis drugs, hindering genotypic-phenotypic comparisons for other drugs. The effectiveness of MDR-TB treatment depends on a combination of bactericidal and sensitive regimens. However, progress is impeded by the toxicity and low bactericidal activity of drugs like linezolid, prothionamide, and cycloserine, which necessitate prolonged treatment durations (up to 24 months). Treatment fatigue or toxicity often leads to non-compliance, increasing the risk of further resistance. In high-burden countries, the lack of effective pDST often results in uncontrolled regimens, further narrowing limited treatment options.

In 2013, the World Health Organization (WHO) first introduced BDQ as a treatment for Multidrug-Resistant Tuberculosis (MDR-TB). When integrated into standardized treatment regimens for both MDR-TB and Extensively Drug-Resistant Tuberculosis (XDR-TB), BDQ has been shown to significantly reduce mortality rates among patients (27, 28). As indicated in Table 3, no resistance was detected for Delamanid (DLM), Bedaquiline (BDQ), or Para-aminosalicylic acid (PAS). These findings are highly encouraging for tuberculosis management in Indonesia, as Bedaquiline and Delamanid are classified as Group A novel anti-tuberculosis drugs, which are critical components in the treatment regimens for Multidrug-Resistant Tuberculosis (MDR-TB) patients. BDQ inhibits MTB ATP synthase, while DLM inhibits mycolic acid cell wall synthesis (29).

Fluoroquinolones; Levofloxacin, moxifloxacin, and gatifloxacin are critical components of MDR-TB regimens due to their high bactericidal activity and potential to shorten treatment duration if dosages are optimized. Although only four pre-XDR-TB strains (1.1%) were identified, this finding is clinically significant: fluoroquinolones are a core component of current MDR-TB regimens, and resistance to this class substantially narrows treatment options and raises the risk of progression to XDR-TB. Given Indonesia’s high MDR/RR-TB burden, and the fact that these strains were found across two islands, in both new and retreated patients, this low prevalence should be treated as an early warning sign that warrants sustained molecular surveillance rather than reassurance. Two further mechanisms of antibiotic resistance that may be in charge of this phenomena are efflux pumps and the enzymatic breakdown of drugs (30).

Genomic analysis confirmed that all four pre-XDR isolates carried critical mutations in the *gyrA* gene, specifically at codon 94 (Asp94Ala or Asp94Gly) (Table 5). These non-synonymous mutations involve the substitution of the negatively charged aspartic acid (Asp), which typically facilitates the binding of magnesium-fluoroquinolone complexes to the DNA gyrase enzyme (31, 32).

The substitution of Asp94 with neutral amino acids such as Alanine (Ala) or Glycine (Gly) inhibits the docking of fluoroquinolones (FQs) in the enzyme’s binding pocket, thereby conferring resistance. FQ resistance TB was found to have a mutation primarily at codons 94 and/or 90, as demonstrated by Kabir et al. (33). Notably, while the Asp94Ala mutation is associated with resistance to third-generation FQs like Levofloxacin, the Asp94Gly mutation often extends this resistance to fourth-generation FQs, including Moxifloxacin (33).

It is significant to note that all four isolates of the pre-XDR were obtained from three of the four pre-XDR-TB patients had never smoked and one was in the 15-24-year age group, indicating that transmission is not confined to classical risk groups and may already be occurring within the community.

Drug-resistant TB can have a negative influence on treatment results for patients in Indonesia, however we can lessen its effects by identifying the variables that contribute to antibiotic resistance in MTB and addressing this issue proactively. Enhancing the identification of drug-resistant strains of MTB, ensuring suitable treatment regimens, encouraging treatment adherence, and fortifying infection control measures are all critical in the fight against antibiotic resistance in the disease. In order to remain abreast of the changing patterns of MTB resistance in Indonesia, research into novel antibiotics and treatment approaches will be essential.

Taken together, the west-to-east gradient in *M. tuberculosis* lineage composition observed across Indonesia underscores the importance of incorporating molecular epidemiological data into national tuberculosis surveillance frameworks. The detection of Lineage 3 for the first time in Indonesia, however at low frequency, signals the importance of sustained genomic surveillance to monitor emerging lineage introductions, particularly given Indonesia’s role as a major maritime hub. Understanding the geographic clustering and boundaries of distinct lineages can inform targeted public health interventions, facilitate the identification of cross-regional transmission events, and contribute to the broader understanding of *M. tuberculosis* population structure in archipelagic Southeast Asia (10). Future studies should aim to integrate lineage data with whole-genome sequencing and patient mobility data to further elucidate transmission dynamics across this geographically fragmented yet epidemiologically interconnected region (20, 21).

### Limitation

There are limitations to our survey. Due to budget constraints, WGS was not done for all RIF-susceptible participants and pDST was not performed for all participants.

This survey represents the largest genomic collection of drug-susceptible and drug-resistant strains from the country and also marks the first time WGS results have been included in the DRS.

## Data Availability

The datasets presented in this study can be found in online repositories. The names of the repository/repositories and accession number(s) can be found in Supplementary file 3.
