# Supplementary material for: Genomic profiling and drug resistance patterns of Mycobacterium tuberculosis in Indonesia: whole-genome sequencing of isolates from the 2017–2018 national Tuberculosis Drug Resistance Survey
Source: Front Public Health. 2026 Oct 27;14:1844150. doi: 10.3389/fpubh.2026.1844150 (PMC13748355; doi:10.3389/fpubh.2026.1844150)
Supplement: Supplementary file 1 [file Supplementary_file_3.docx]

**Data Availability**

WGS data for four pre-XDR *M. tuberculosis* strains in the National Center for Biotechnology Information (www.ncbi.nlm.nih.gov). Sequence Read Archive (SRA) under BioProject accession number PRJNA950554.

| [**Accession**](https://dataview.ncbi.nlm.nih.gov/) **Number** | **Title** | **BioSample** | [**Library**](https://dataview.ncbi.nlm.nih.gov/) **ID** |
| --- | --- | --- | --- |
| [SRR29659248](https://dataview.ncbi.nlm.nih.gov/object/SRR29659248) | WGS of *Mycobacterium tuberculosis*: culture | [SAMN42182043](https://dataview.ncbi.nlm.nih.gov/object/51358688) | R3619 |
| [SRR29659249](https://dataview.ncbi.nlm.nih.gov/object/SRR29659249) | WGS of *Mycobacterium tuberculosis*: culture | [SAMN42182042](https://dataview.ncbi.nlm.nih.gov/object/51358687) | R3618 |
| [SRR29659247](https://dataview.ncbi.nlm.nih.gov/object/SRR29659247) | WGS of *Mycobacterium tuberculosis*: culture | [SAMN42182044](https://dataview.ncbi.nlm.nih.gov/object/51358689) | R737 |
| [SRR29659250](https://dataview.ncbi.nlm.nih.gov/object/SRR29659250) | WGS of *Mycobacterium tuberculosis*: culture | [SAMN42182041](https://dataview.ncbi.nlm.nih.gov/object/51358686) | R475 |
